# Sweetpotato seed exchange systems and knowledge on sweetpotato viral diseases among local farmers in Acholi Sub Region-Northern Uganda

**DOI:** 10.5897/AJAR2018.13384

**Published:** 2018-11-08

**Authors:** Godfrey Wokorach, Hilary Edema, Richard Echodu

**Affiliations:** 1 Gulu University Biosciences Research Laboratories, P. O. Box 166, Gulu, Uganda; 2 Department of Biology, Faculty of Science, Gulu University, P. O. Box 166, Gulu, Uganda

**Keywords:** Sweet potato, seed exchange, local farmers, farming practices, vine selling

## Abstract

Viral infections greatly limit sweetpotato yields. Good farming practices are critical for effective disease management. 383 Ugandan farmers were surveyed to document symptom incidence, crop-management practices, and buyer preferences. Results showed that 89.27% farmers grow sweet potatoes yearly and 62.76% of these farmers were female. A total of 56.83% farmers obtained vine seeds from their previous gardens, 25.85% from neighbours, and 12.20% purchased. Only ~8% of sellers and ~4% of buyers were selected for disease-free materials. None of the farmers who used vine-cutting knives sterilised them. Almost half of farmers (47%) observed whitefly or aphids but most were unaware they are viral vectors. Most farmers (77%) observed viral symptoms, but few (<2%) recognised them as infections. Insufficient knowledge of sweetpotato viruses and their vectors is common and increases the risk of spread. Practices like vine selling, sharing of vines coupled with insufficient knowledge on sweet potato viruses and its vectors among farmers increase the risk of virus spread among different farms.

## INTRODUCTION

Sweetpotato (*Ipomoea batatas* (L.) Lam.), is considered to be the third most important root crop after cassava and potato (Mukasa et al., 2003; Kashif et al., 2012). In Uganda, the crop is grown both at subsistence level for home consumption and for sale in local markets (Aritua et al., 2007; Kivuva et al., 2014). Sweetpotato is an excellent crop for small household farmers because it yields relatively well in poor soil, provides an important source of carbohydrates, and its tubers can remain in the soil for extended periods thus providing a continuous food source. The production of orange-fleshed sweetpotato is being particularly encouraged among local farmers because it contains beta-carotene, which is a precursor of vitamin A, and is thus one of the cheapest means of alleviating vitamin A deficiency (Kivuva et al., 2014).

In East Africa, sweetpotato production is concentrated around Lake Victoria and Uganda is among the largest producers of sweetpotato in Africa (Karyeija et al., 1998; Byamukama et al., 2004). Sweetpotato is the second most important root crop in Uganda and is grown in almost every district (Mukasa et al., 2003). In many areas of East Africa, where sweetpotato production is high, production has not yet attained its full potential (Kivuva et al., 2014); the high production of sweetpotato in Uganda is attributed to the large cultivated area rather than high yield (Byamukama et al., 2004). The total area of sweetpotato in Uganda is estimated at 4,440,000 ha (Uganda Bureau of Statistics, 2010). Given production potential of 25 t/ha for virus free vines (Clark et al., 2012), Uganda’s expected production capacity is predicted to be about 1.11 × 10^8^ t. The yield attained has, however, been lower than expected ranging from 1.8 × 10^6^ t in 2009 to 2.55 × 10^6^ t in 2011 (Caliskan et al., 2007; Uganda Bureau of Statistics, 2010; Okonya and Kroschel, 2014). Yields of sweetpotato below production potential can be a result of either abiotic or biotic constraints (Kivuva et al., 2014). Sweetpotato yield loss due to viral infections ranks second after weevil infestation (Opiyo et al., 2010). Co-infection of sweetpotato plant with sweetpotato feathery mottle virus and sweetpotato chlorotic stunt virus produce severe disease syndrome known as sweet potato virus disease (SPVD) (Gutiérrez et al., 2003). SPVD is the major sweet potato disease in East Africa which cause yield loss of up to 98% (Mukasa et al., 2003).

Vegetative propagation of sweetpotato remains the most important mechanisms for the spread, survival and transmission of sweet potato viruses from generation to generation (Adane, 2010). Farmers often have the tendency to leave their sweetpotato vines from previous seasons to sprouts and provide planting materials for next season. As result, this promotes the accumulation of the viruses each season (Karyeija et al., 1998). The practices increase the chances of distribution of viruses over wider areas.

In Uganda, most farmers obtained planting materials from previous own sweetpotato farm (Bashaasha et al., 1995). However, some farmers obtained vines from neighbours’ sweetpotato garden usually for free. Under extreme condition farmers reported buying of sweetpotato vines (Bashaasha et al., 1995). Recently non-governmental organisation like world vision and harvest plus were involved in sweetpotato vine multiplication and distribution among local farmers (Gibson, 2013). As result informal vine multiplication dealers were established by the organisations to increase accessibility of orange flesh cultivars among local sweetpotato farmers (Gibson, 2013).

Northern Uganda suffered from nearly two decades (1986 to 2006) of war, which led to breakdown in the agriculture sector as most people were settled in camps for internally displaced people. Since 2006, people have resettled in their former villages and struggled to recover from effects of the war by engaging in agricultural activities to improve their livelihoods. However, most farmers in Africa use traditional practices that unknowingly enhance the spread of crop diseases (Karyeija et al., 1998; Kivuva et al., 2014). Viral diseases are the most difficult to manage and therefore good farming practices are critical for effective disease management, but there is little information on farming practices for sweetpotato in this region. As previous studies on sweetpotato virus in Uganda did not extensively cover northern Uganda due to war at that time. As result little information is available on sweetpotato farming practices in Acholi sub region which was at the epicentre of the two decades of insurgency. The aim of this study was to explore the different farming practices of local farmers in Northern Uganda that promote transmission and spread of sweetpotato viruses among farmers’ fields. A cross-sectional survey was performed in three districts and 383 questionnaires were administered.

## MATERIALS AND METHODS

The survey was conducted in Gulu, Kitgum and Lamwo out of the seven districts comprising the Acholi sub-region in Northern Uganda. Kitgum and Gulu were chosen because they were the two pioneer districts for production of sweetpotato in Acholi. It was assumed that the farming practices used for growing sweetpotato in all newly formed districts would not deviate much from the two districts (that is, Kitgum and Gulu) from which they were derived. The Lamwo district borders South Sudan and there is frequent interaction between the two populations. Lamwo was therefore the third logical addition to explore any hybrid farming practices that may have arisen from people moving between Lamwo and South Sudan. Northern Uganda is located at about 1100 m above sea level. The area experience unimodal pattern of rainfall that last from April to October every year. The temperature varies from 26 to 29°C between April and November. The temperature can rise to 37°C during dry season usually from December to March

A total of 383 farmers in the three districts were interviewed. The number of farmers interviewed was determined using a statistical formula described by Krejcie and Morgan (1970). The population of smallholder farmers who grow sweetpotato in Northern Uganda was estimated at 100,512 (Uganda Bureau of Statistics, 2010). The C-survey 2.0 (UCLA/Fogarty AIDS International Training and Research Program, Los Angeles, CA, USA) was used to generate and assign random numbers to the complete list of sub-counties in each district. Two sub-counties were then selected using a random-numbers table. Questionnaires were administered in person to farmers in these sub-counties. A total of 152 questionnaires were administered in Gulu as it is a bigger producer of sweetpotato than the other two districts, with a production capacity of 61,732 t annually (Uganda Bureau of Statistics, 2010). Questionnaires were administered to 124 farmers in Kitgum and 107 in Lamwo. The farmers were presented with picture of plants showing sign of viral infections (purple chlorotic spot on leaf, yellow chlorosis of the leaves, vein clearing, leaf mottling, and leaf mosaic) and then asked questions to assess their understanding. Similarly, farmers were presented with pictures of *Bemisia tabaci* (Gennadius) or *Myzus persicae.*

Questionnaires were administered to farmers with sweetpotato gardens and to farmers who sold vines. Those farmers who sold vines were required to answer section 3 of the questionnaire, which contained questions on how vines were sold. The study was approved by the Department of Crop Protection unit under the Ministry of Agriculture Animal Industry and Fishery with file number CCP/95. Oral informed consent was obtained for every individual farmer interviewed. The questionnaires were entered into the database created in EPI info 7 (CDC, Atlanta, USA). Raw data were exported to Microsoft excel for data validation. The validated data were uploaded back to EPI info 7. Frequency of response to each question was computed and expressed as percentage. The data were presented inform of table and figure.

## RESULTS

### Sweetpotato vine exchange system

Majority of farmers, 63.71% interviewed were female and male were only 36.29% (Supplementary Table 1). Most farmers, 89.3% grew sweetpotato every year; the other 10.7% skipped some years (Table 1). Of the farmers who grow sweetpotato every year, 62.76% (Table 1) were female. Females were 1.43 times (Supplementary Table 2) more likely to grow sweetpotato yearly than males. Sources of sweetpotato vines for most farmers 75.25% where near their homestead and only 5.10% farmers move to another districts sourcing sweetpotato vines for planting (Supplementary Table 3). Most of the farmers surveyed 56.83% (Table 1) obtained vine cuttings from their previous gardens/fields (volunteer vines). Meanwhile, 12.2 and 5.12% of farmers obtained vines from market and their relatives, respectively (Table 1). Females who grow sweetpotato are 1.94 times (Supplementary Table 4) more likely to buy sweetpotato vines for planting compared to male sweetpotato farmers.

**Table 1:** Frequency that local farmers grow sweetpotato and the sources of their vines.

Variable	Frequency	Percentage
**Frequency that farmers grow sweetpotato**		
Yearly	341	89.27
Not yearly	41	10.73
Total responses	382	100.00
**Grow sweetpotato yearly**		
Male	127	37.24
Female	214	62.76
Total	341	100
**Sources of sweetpotato vines**		
Own farm	233	56.83
Neighbour’s farm	106	25.85
Market	50	12.20
Relative’s farm	21	5.12
Total responses	410	100

In total, 21% of farmers travelled out of their district to obtain planting materials. A higher proportion of farmers who source vines outside their districts were from Kitgum district and followed by farmers from Lamwo district. A total of 78.72% of farmers in Kitgum and 40% of farmers in Lamwo district who travelled to obtain vines from another district reported Gulu district as their source. Female farmers were 0.69 time less likely to source sweetpotato planting materials beyond the boundary of their district of residence compared with male farmers. Of the farmers interviewed, 82.5% deliberately selected sweetpotato varieties to plant, whereas only 17.5% did not select vines (Table 2). The majority of farmers (33.9%) favoured high-yielding vines, 31.3% liked early maturity and only 6.0% preferred healthy vines (Table 2).

**Table 2:** Criteria used by farmers when selecting sweetpotato vines.

Variable	Frequency	Percentage
**Select vines for planting when cutting**		
Yes	316	82.51
No	67	17.49
Total responses	383	100
**Qualities of vines that farmers prefer**		
High yield	130	33.33
Mature quickly	120	30.77
Other (specify)	34	8.72
Healthy vines	23	5.90
Taste preference	23	5.90
Broad leaves	20	5.12
Disease tolerance	15	3.85
Drought tolerance	13	3.33
Delayed rotting of tubers in field	8	2.05
Ease of access to vines	4	1.03
Total responses	390	100

### Sweetpotato harvest practices

A total of 59.8% farmers were unable to complete harvest of their sweetpotato from old gardens before the new planting season commenced in which they expected to plant a new crop. By contrast, 40.2% farmers completed harvest before the new season commenced. It was found that 97.0% farmers preferred piecemeal harvest, compared to 3.0% of farmers who harvested by clearing the garden all at once. About one-fifth (21%) of farmers preferred intercropping sweetpotato compared to 79.0% who grew sweetpotato as a monocrop.

A total of 83.0% of farmers preserved vines to plant in the new season, in contrast to only 17.0% who never preserved vines for the new season. There were 66.1% of farmers who kept vines and preferred to leave vine remnants in their gardens, to sprout spontaneously when rain commenced in a new rainy season (Table 3). In addition, 16.2% of farmers preferred growing vines in moist areas during the dry season to prevent them from desiccating and only 3.7% planted vines in protected areas to prevent animals from destroying them. A total of 99.4% of farmers used a knife to cut sweetpotato vines and, surprisingly, none of these farmers sterilised their knife during vine cutting. Only 9.2% of farmers reported that they cleaned their knife with non-disinfectant agent like clothes, stones or soap (Table 3).

**Table 3:** Methods for preserving vines and tools used for vine cutting

Variable	Frequency	Percentage
**Preserve vines for planting**		
Yes	318	83.03
No	65	16.97
Total responses	383	100
**Methods for preserving vines**		
Leaving them to sprout in garden (volunteer vines)	253	66.06
Growing them in moist place	62	16.19
Growing them in a conserved area	14	3.66
Keeping them in refrigerator	0	0.00
No response	54	14.09
Total responses	383	100
**Tools used for cutting vines**		
Knife	381	99.48
Others (specify)	0	0.00
Sickle	0	0.00
Scissors	0	0.00
No response	2	0.52
Total responses	383	100
**Clean tools during vine cutting**		
Yes	35	9.21
No	345	90.79
Total responses	380	100
**Materials used for cleaning vine-cutting tools**		
Cotton	1	3.23
Files	1	3.23
Piece of cloth	8	25.80
Stones	3	9.68
Water	2	6.45
Water and soap	15	48.38
Water and stones	1	3.23
Total responses	31	100

## Vine selling

Only 5.48% of farmers have ever engaged in selling of sweetpotato vines. Most farmers who sold vines (71.4%), came from Gulu; 19.0% were from Kitgum and 9.5% were from Lamwo (Table 4). Of the 21 vine sellers, 19 reported that buyers generally request vines with the attributes they liked the most. Only two sellers reported they had buyers who bought any kind of vine. About half (12 of 21) of sellers recorded that buyers preferred vines with high-yield attributes; seven sellers said their buyers preferred vines that matured rapidly (Table 4). Only one vine seller reported that buyers selected vines based on the taste of tubers and only two sellers reported that buyers preferred healthy vines. Of the 21 vine sellers, 15 preferred selling vines with high-yield traits, four opted for vines that were easy to get and only two opted to sell disease-free vines (Table 4).

**Table 4:** Vine sellers and qualities of vines preferred by buyers based on a survey of 21 sellers.

Variable	Frequency	Percentage
**Vine selling per district**		
Gulu	15	71.43
Kitgum	4	19.05
Lamwo	2	9.52
Total responses	21	100
**Inquiry on vine quality being sold by sellers**		
Yes	19	90.48
No	2	9.52
Total responses	21	100
**Qualities of vines from sellers**		
High yield	12	52.17
Early maturity	7	30.43
Others	2	8.70
Disease free	1	4.35
Taste preference	1	4.35
Total responses	23	100
**Qualities of the vines that sellers take to market**		
High yield	15	60.00
Easy to obtain	4	16.00
Variety	2	8.00
Disease free	2	8.00
Drought resistant	1	4.00
Taste preference	1	4.00
Total responses	25	100

### Knowledge about sweetpotato viruses and their vectors

About half (46.7%) of farmers had observed *Bemisia tabaci* (Gennadius) or *Myzus persicae* (aphid) in their sweetpotato gardens compared with 53.3% who had never observed the vectors in their gardens. Most (78.7%) of the interviewed farmers were unaware of the viral threats associated with whitefly and aphids. Only 14.1% of farmers reported use of pesticides in their sweetpotato gardens. Female farmers were 0.73 times (Supplementary Table 5) less likely to know the vectors of sweetpotato viruses compared to male farmers and thus females were 0.70 time (Supplementary Table 6) less likely to assess for the presence of the vectors compared with male farmers.

Most (77%) sweetpotato farmers said they had seen symptoms of infection as presented to them in the questionnaire pictures. A small number of farmers (14.3%) had no idea of the possible cause of symptoms they had seen in sweetpotato plants, 15.15% attributed the symptoms to disease infection and 13.5% to insect pest infestation (Table 5).

**Table 5:** Farmers’ beliefs about what causes abnormal appearance of sweetpotato vines. Abnormal appearance refers to all symptoms of viral infection and includes curled leaves, mosaic leaves, vein clearing, mottled leaves, yellow chlorosis, necrotic spots on leaves, purple chlorosis and stunted plants.

Causes of abnormal appearance of sweetpotato	Frequency	Percentage
Ash deposited in field	1	0.34
Caterpillars	18	6.06
Excess vines in the garden	1	0.34
Heavy rain	1	0.34
Infection	45	15.15
Insects	40	13.47
Millipedes	15	5.05
Mixture of varieties	4	1.35
Nematodes	33	11.11
No idea	55	18.52
No response	4	1.35
Old age	7	2.36
Pests	16	5.39
Soil infertility	10	3.37
Sun burn	36	12.12
Viruses	5	1.68
Weeds	6	2.02
Total responses	297	100.00

**Figure 1:**
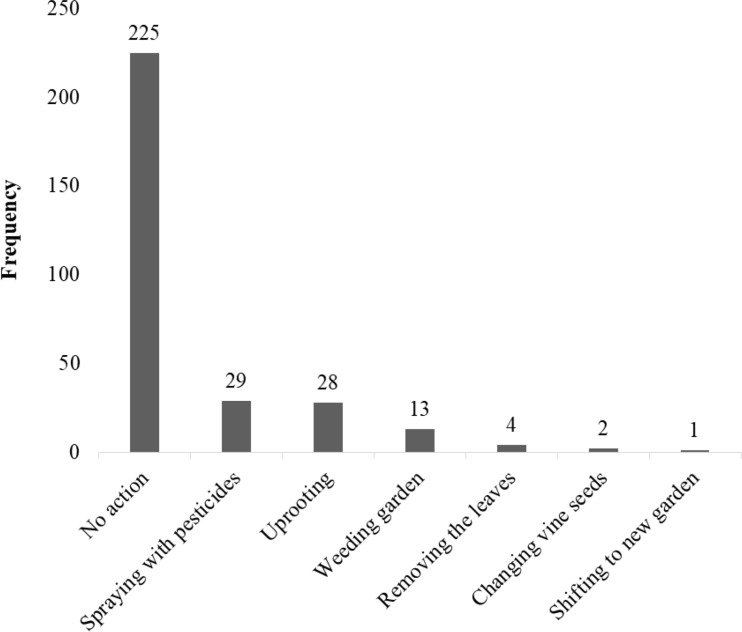
Management strategies taken by 383 surveyed farmers when sweetpotato plants exhibited symptoms of viral infection.

Only 31% of farmers regularly checked vines for symptoms of sweetpotato viruses. Most (74.5%) of farmers took no action even when they observed virus-like symptoms; 9.6% sprayed their gardens with some kind of pesticide once such symptoms appeared (Figure 1).

## DISCUSSION

This study confirmed that the majority of sweetpotato farmers were women, as was reported in other studies (Karyeija et al., 1998; Kivuva et al., 2014). It was reported that men tend to engage more in production of crops with high economic value (such as *Sesamum indicum*) that fetch higher prices in the market than sweetpotato in order to meet the financial demands of the family (Kivuva et al., 2014). Men are more likely than women to hold salary jobs in the urban and semi-urban centres, which tend to keep them away from their villages and their farms (Kivuva et al., 2014). Regarding sweetpotato production, men may only participate in heaping mounds whereas most of the other activities (e.g., vine gathering, planting and harvesting) are done by women and children. Reports show that women have limited access to agriculture extension services, which has serious implications for dissemination of knowledge and the fostering of good farming and production practices (Karyeija et al., 1998; Okonya and Kroschel, 2014).

Most farmers grow sweetpotato yearly, indicating that it is a priority crop for most farmers, especially for women who grow it for household consumption. Sweetpotato can thrive in poor soils, requires few inputs and can be harvested many times within a season (Clark et al., 2012), making it preferred by rural farmers as a safeguard against extreme food shortages. Some farmers also use sweetpotato as a source of income, especially for women who sell the tubers in the local markets. Some farmers have shifted to growing sweetpotato because of the continued devastating effect of cassava mosaic disease and cassava brown streak disease on cassava production in the region (Scott et al., 1997; Kumakech et al., 2013).

Most farmers get their sweetpotato vines near their homes from their own, neighbours’ or relatives’ gardens (Table 1). The practice of sharing sweetpotato vines among farmers is very common and this tradition was previously reported in among different sweetpotato growing communities (Karyeija et al., 1998). The practice has been implicated as the major contributing factor in the spread of sweetpotato viruses among distant farms because the exchange of vines among farmers not only occurs within villages or sub-counties but also among farmers in different districts and countries. It was found that cross-boundary movement of vines among different districts was common. Movement of vines is greatly facilitated by the extreme shortage of vines or the presence of unique traits that are attractive to farmers. We found a high degree of movement of sweetpotato vines from Gulu to the neighbouring districts of Kitgum and Lamwo. This study suggests that it will be important to perform extensive and frequent virus surveillance in Gulu specifically because it is an important centre for supply and distribution of sweetpotato vines in the northern part of Uganda. The results also suggest that Gulu could be a major centre for distribution of sweetpotato viruses in Northern Uganda.

Although no farmer reported obtaining sweetpotato materials from districts in Central and Western Uganda where the sweetpotato virus burden is reportedly very high (Aritua et al., 2007), we cannot rule out exchange of vines among farmers in the three districts of the present study with those in other regions of Uganda. Additionally, there was evidence of imported vines in the present study; one person reported getting vines from Democratic Republic of Congo. Surveillance of the movement of sweetpotato materials across country borders is very important because such movements can lead to the introduction of additional viruses and viral strains, including those with more serious effects (Mukasa et al., 2003; Aritua et al., 2007).

The preferences for most farmers to grow specific sweetpotato varieties were driven by local preferences about vine varieties, mainly high yield and early maturity rather than the health status and taste preference (Table 2). To minimise the spread of sweetpotato viruses, farmers first need to select vines that are visually healthy. Of course, not all vines that look healthy are virus-free (Aritua et al., 2007), so additional practices must be in place. The most effective choice is to use virus-free germplasm cuttings from seed multiplication centres or tissue culture labs; however, access to such services is very hard for local farmers in rural settings and the number of vines supplied is insufficient for all farmers. Farmers need to be trained in viral symptom identification so that they can select healthy vines.

In addition to growing sweetpotato in wetlands during the dry season, most farmers preserve their vines by allowing remnants of vines of the previous season to sprout (so-called “volunteer vines”). Regardless of the techniques used for preservation of these vines, the consequences of this practice are very important. This practice allows cyclic use and reuse of the same planting materials for an extended number of years, which may favour accumulation of high virus titre within infected plants. Such vines may be distributed to wider areas by sale and sharing among friends, neighbours and relatives. Accumulation of virus titre within infected plants degenerates the potential of the plants to achieve high yields. The preferred cultivars or varieties (which are widely shared because of their high yields) will increasingly experience declined yields as a result of virus-induced degeneracy. Such cultivars with virus induced low yield will be widely rejected and their production abandoned among local farmers. This will lead to narrower pool of genetic resources and potentially the extinction of some cultivars. Despite the challenges with the “volunteer vines” method of vine preservation, this practice does result in limiting the influx of vines from other virus-prone areas, which is the case in Gulu where few farmers look for vines from other districts, compared to Kitgum where there is minimal preservation of vines.

Piecemeal harvesting is a common practice among most sweetpotato farmers in Africa because the tubers do not readily spoil (Karyeija et al., 1998). In this area, farmers usually use a stick to excavate mounds and remove the tubers. They usually take one or two tubers from each mound and leave immature tubers to continue growing for the next harvest. Farmers can therefore harvest sweetpotato on a daily or weekly basis and it can be up to 5 to 6 months before harvest is complete, which in most cases is during the dry season. The attraction of piecemeal harvest is that it regularly supplies tubers for an extended period and provides a sustainable food source for the household. However, the practice also maintains plants for long in the fields which often overlap into new planting seasons. Usually vines from such old fields form the first source of planting materials among local farmers. If they are infected, these plants become the source of infection in new sweetpotato fields.

Most farmers use knives for cutting new vines to be transferred to their new gardens or to take to markets (Table 3). The importance of sterilising cutting tools is emphasised in the sweetpotato production manual (Dennien et al., 2013); however, it is clear from our study that farmers lack this vital information. Farmers usually use a single knife to cut as many vines as possible in one round of sweetpotato vine gathering. This practice provides a substantial risk of the knife becoming contaminated and then spreading viruses to all subsequent cuttings. Sterilisation measures are requisite for effective control. Although we could not find any reports of sweetpotato virus being spread through contaminated cutting tools, mechanical transmission of some viruses to virus-free vines by inoculation with sap from infected plants has been demonstrated (Domola, 2003; Wosula et al., 2012). Use of contaminated tools has been implicated in the spread of other plant viruses, especially in citrus during pruning (Garnsey and Whidden, 1971). If this is the case with sweetpotato, then the scope for viral spread and transmission is very broad and the risk is high.

Vine selling is accelerated by extreme shortage of sweetpotato vines because of the prolonged dry spell in the dry season, which scorches most vines except those in wetlands. Most farmers have limited access to wetlands where they can preserve vines during the dry season. Conditions are worse in Lamwo and Kitgum, which experience greater heat during the dry season than Gulu. There are relatively more people who grow sweetpotato during the dry season in wetland in Gulu than in Lamwo and Kitgum. Farmers grow sweetpotato during the dry season for three main reasons: to preserve vines for themselves, to preserve vines for sale at the start of the rainy season and for sale of tubers during the period of shortage. Gulu district therefore becomes a major centre where there are vine sellers, and most farmers can get sweetpotato vines for the new rainy season. Sweetpotato vines that are sold in the open market by farmers do not undergo a process of virus-free certification and the chances of buying infected vines are very high. In addition, most farmers who buy such vines tend to focus on traits such as early maturity, yield and tuber taste; they do not consider the disease status of vines.

*B. tabaci* and *M. persicae* are the two main insect vectors that spread sweetpotato viruses and aid in co-infection of sweetpotato plant by two or more viruses. The present study reveals that farmers have insufficient knowledge concerning these vectors. This limited knowledge can be attributed to the rarity of vectors within their farms or inadequate sensitisation of farmers to the presence and potential threat of these vectors. Reports indicate that women have limited access to essential agricultural information such as that concerning vectors and pests (Okonya et al., 2014), and women are the predominant sweetpotato farmers in this area. It has also been reported that extreme weather during the dry season does not favour rapid multiplication of the vectors and consequently their populations drop in this area, which experiences a prolonged dry spell during the dry season, which may account for a rarity of vectors. Further studies should be performed to adequately assess the populations of these two vectors.

Only a few farmers reported the use of pesticides to control insect pests. Similarly, Bashaasha et al. (1995) found that only a few sweetpotato farmers in Gulu and other districts in Uganda use pesticides in controlling insect pests of sweetpotato. Pesticide or insecticide application would reduce the population of whitefly and aphids and subsequently reduce spread viruses among sweetpotato plants (Opiyo et al., 2010). However, the costs associated with pesticides results in most local farmers adopting less expensive methods; for example, some farmers slash off the dense vegetative cover of sweetpotato to remove the food source for major pests such as caterpillars. Thus, vectors could potentially be regulated by such natural means as predators, parasitoids and prolonged dry spells, rather than farmer intervention, but more studies need to be done before management suggestions can be made.

Most farmers are unaware of sweetpotato virus diseases, the symptoms of virus infection and the burden associated with viruses. Limited knowledge of sweetpotato viruses is not unique to Northern Uganda but is commonly reported among farmers in other parts of Africa (Domola, 2003). Limited knowledge was evident in the present study by many farmers mistaking damage on sweetpotato by insect pests to be virus symptoms. Such inadequate knowledge has implications for the management and control strategies farmers take on diseased plants. Most farmers tend to keep diseased vines because the vines have known good attributes. Additionally, some of the actions ordinary farmers take (Figure 1) are insufficient to curtail spread of sweetpotato viruses because they do not focus on destroying but rather maintaining the diseased plants. Thus, the vines continue to be used as planting materials, maintaining the virus for longer and potentially acting as a source of further infection. There is thus an urgent need to train farmers on symptom identification and possible measures concerning vines that look infected.

## Conclusions

Exchange of sweetpotato planting materials among local farmers was high. In most cases such exchange occurs among neighbourhoods, relatives or farmers from different districts by sale of vines. The exchange of vines at different levels among local farmers risks spreading infected vines to wider geographical areas within the country. Additionally, limited knowledge of the symptoms of sweetpotato viruses among local farmers results in the use of infected vines for an extended period and the planting of new infected vines into their fields. This results in the perpetuation of virus-infected sweetpotato in the production cycle. Thus, there is a great need for local farmers in this area to be sensitised to the presence of viruses, be familiar with the symptoms of virus manifestation and understand the extent of damage caused to sweetpotato production. It is also important that local farmers be taught and encouraged to use phyto-sanitation measures such as uprooting diseased plants and planting visually healthy vines.

## Figures and Tables

**Supplementary Table 1:** Socio demography of the respondents.

Variable	Frequency	Percentage
**Age (years)**		
11-20	15	3.93
21-30	132	34.55
31-40	112	29.32
41-50	61	15.97
51-60	28	7.33
61-70	25	6.54
71-80	5	1.31
81-90	4	1.05
Total	382	100
**Marital status**		
Single	45	11.87
Married	286	75.46
Divorced	30	7.92
Others (widow, widower and co-habiting)	18	4.75
Total	379	100
**Sex of respondents**		
Male	139	36.29
Female	244	63.71
Total	383	100

**Supplementary Table 2:** Odds of growing sweetpotato yearly between male and female farmers

How often you grow sweetpotato	Gender		Odds ratio		

	Male	Female	Odds estimate	Upper odds	Lower odds
Yearly	27	214			
Not yearly	2	29	1.4342	0.7068	2.9104

**Supplementary Table 3:** Distance of vine source from farmers gardens.

Variable	Frequency	Percentage
**Distance of vine source from farmer’s garden**		
Near home	295	75.25
Another village	64	16.33
Another district	20	5.10
Another sub-county	13	3.32
Total	392	100

**Supplementary Table 4:** Odds of buying vines between female and male.

Buy vines from market	Gender		Odds ratio		

	Female	Male	Odds estimate	95% Upper interval	95% Lower interval
Yes	41	13			
No	203	126	1.95	1.009	3.796

**Supplementary Table 5:** Odds of knowledge on vectors between male and female.

Knowledge on vectors	Gender		Odds ratio		

	Female	Male	Odds estimate	95% Upper interval	95% Lower interval
Yes	107	72			
No	137	67	0.73	0.478	1.1038

**Supplementary Table 6:** Odds of farmers who asses presence of vectors on gardens.

Asses presence of vectors on garden	Gender		Odds ratio		

	Female	Male	Odds estimate	95% Upper interval	95% Lower interval
Yes	46	35			
No	195	104	0.70	0.425	1.1556
